# Editorial: 4D printing and 3D printing in robotics, sensors, and actuators manufacturing

**DOI:** 10.3389/frobt.2022.1110571

**Published:** 2022-12-13

**Authors:** Ali Zolfagharian, Mahdi Bodaghi, Antoine Le Duigou

**Affiliations:** ^1^ School of Engineering, Deakin University, Geelong, VIC, Australia; ^2^ Department of Engineering, School of Science and Technology, Nottingham Trent University, Nottingham, United Kingdom; ^3^ University Bretagne Sud, UMR CNRS 6027, Bionics group, Lorient, France

**Keywords:** 4D printing, soft robotics, sensors, actuators, additive manufacturing

Recent advances in additive manufacturing (AM) techniques, particularly three-dimensional (3D) printing and four-dimensional (4D) printing have enabled the production of different parts of robots, including sensors and actuators, with high multifunctionality, compliance, and manufacturing flexibility unlike in traditional approaches (Bodaghi and Zolfagharian, 2022). Based on pre-programmed architectures that are often inspired by biological structures, 4D-printed robots and soft robots are adaptive mechanisms responding to external stimuli, like temperature, force, electricity, light, and humidity made with polymeric based composites (Le Duigou and Correa, 2022). Indeed, the autonomous actuation offered by 4D printing envisages frugal engineering with robots without wire nor motor. They can be applied in a wide range of applications, including medical assisted robots, automotive, aerospace, microfluidics, tissue engineering, drug delivery, and wearable electronics (Zolfagharian et al., 2022). The design and prediction of behavior and control of these products is yet a complex problem requiring the multiphysics formulation where machine learning and deep learning techniques have been promising. This Research Topic aimed to promote the dissemination of recent advances in AM of soft robots, including sensors, actuators, metamaterials, morphology control and novel design strategies. This presented *via* five original research articles jointly made by involvement of 22 active academics in the field from Austria, Germany, Switzerland, Japan, Australia, the United Arab Emirates, and the United States.

In the opening article, Dämmer et al. from Johannes Kepler University Linz, Austria, in collaboration with Bram Vanderborght, in Advanced Development Control and Robotics, Festo SE and Co. KG, Germany, have presented a rotary pneumatic actuator additively manufactured *via* inkjet printing. They demonstrated that the complex structural behavior of the actuator’s elastomeric bellows structure can be predicted by Finite Element (FE) simulation. The simulations presented in their work contributed to the materials science of inkjet-printed elastomers by demonstrating the use of a hyper viscoelastic material model for estimating the deformation behavior of a prototyped robotic component. The results contribute to the long-term goal of additively manufactured and pneumatically actuated lightweight robots.

Georgopoulou et al. from Empa–Swiss Federal Laboratories for Materials Science and Technology, Switzerland, and the scientists in Vrije Universiteit Brussel, Belgium, have published their work on the development of an open-source soft robotic gripper to evaluate the fused deposition modeling (FDM) printing of thermoplastic polyurethane (TPU) structures with integrated strain sensing elements in order to provide some guidelines for the material selection when an elastomer and a soft piezoresistive sensor are combined. Such soft grippers, with integrated strain sensing elements, were successfully printed using a multi-material FDM 3D printer. Their findings show that the *in situ* printed strain sensing elements on the soft gripper were able to detect the deformation of the structure when the tentacles of the gripper were open or closed. The sensor signal could differentiate between the picking of small or large objects and when an obstacle prevented the tentacles from opening. The correlation between TPU Shore hardness, which was used for the gripper body, and the sensitivity of the integrated *in situ* strain sensing elements revealed that material selection has a significant impact on the sensor signal. This study can be an example for guiding the selection of material combinations for soft robotic systems produced with FDM.

Shintake et al. from the University of Electro-Communications, Japan, demonstrates a method to monolithically fabricate stacked dielectric elastomer actuators (DEAs) without alternately stacking the dielectric and electrode layers in their article. They have investigated the feasibility of the method *via* the fabrication and characterization of simple monolithic DEAs with multiple electrodes. The fabricated actuators are characterized in terms of actuation stroke, output force, and frequency response. In the actuators, polydimethylsiloxane (PDMS) and eutectic gallium–indium (EGaIn) are used for the elastomeric matrix and electrode material, respectively. Microfluidic channels are realized by dissolving a 3D-printed part suspended in the elastomeric structure. The experimental results show the successful implementation of the proposed method and the good agreement between the measured data and theoretical predications, validating the feasibility of the proposed method.

In the fourth article, Tawk et al., in a joint work between University of Wollongong in Australia and the United Arab Emirates, have presented the application of metamaterials in the design of soft robotic grippers. They created a 3D-printed modular soft gripper with highly conformal soft fingers made of positive pressure soft pneumatic actuators and a mechanical metamaterial. The fingers of the soft gripper, along with the mechanical metamaterial, which integrates a soft auxetic structure and compliant ribs, were 3D printed in a single step, without requiring support material or postprocessing, using a low-cost and open-source FDM 3D printer and commercially available TPU. The FE simulations accurately predicted the behavior and performance of the fingers in terms of deformation and tip force, thus proving its effectiveness in enhancing the grasping performance of the gripper.

The Research Topic closes with the work of Stanislav et al. from the College of Engineering, Embry–Riddle Aeronautical University, United States. They have studied on the modeling and fabrication of unimorph DEA, a configuration capable of producing large bending deformation and one of the most suitable to be implemented through 3D printing. The proposed model allowed for illustrating the effect of each UDEA layer on actuator performance and obtaining several figures of merit for simple design optimization and material selection. Intending to facilitate research on any 3D printed soft actuators and robotics, this paper demonstrates the fabrication challenges of DEA comprised of commercially available silicones utilizing accessible 3D printing equipment. Materials’ manufacturability, viscosity, and curing are mainly studied to determine their printing handling time. They have validated the developed modeling and fabrication methodologies using 3D printing by testing a unimorph DEA.
